# Novel circuitry and molecular pathogenic mechanisms in Huntington's disease

**DOI:** 10.1186/1750-1326-7-S1-L18

**Published:** 2012-02-07

**Authors:** X William Yang

**Affiliations:** 1 Center for Neurobehavioral Genetics, Semel Institute, Department of Psychiatry & Biobehavioral Studies, David Geffen School of Medicine at UCLA, LA, California, USA

## Background

Huntington's disease (HD) is one of the most common inherited neurodegenerative disorders and is characterized clinically by the mid-age onset of progressive motor, cognitive and psychiatric deficits.

## Methods

HD is caused by a CAG repeat expansion encoding an expanded glutamine repeat near the N-terminus of the huntingtin protein. How mutant huntingtin (mhtt) causes progressive and selective degeneration of striatal and cortical neurons, leading to symptoms of HD, remains unclear. In this presentation, I will describe a novel reductionist approach to use Cre/LoxP conditional Bacterial Artificial Chromosome (BAC) transgenic mouse models expressing full-length mhtt to dissect circuitry and molecular pathogenic mechanisms in HD.

## Results

Our study defines the distinct but synergistic roles of cortical and striatal projection neurons in HD pathogenesis, and uncovers novel evidence for HD being a non-cell-autonomous disease. Finally, we show that phosphorylation of a small mhtt N-terminus domain can act as a molecular switch to suppress HD pathogenesis *in vivo*.

## Conclusion

Together, our studies provide novel mechanistic and therapeutic insights for HD.

